
JOURNAL INFORMATION
==============================
NLM Title Abbreviation: ChemistryOpen
Journal ID: ChemistryOpen
NLM Journal ID: 101594811
Journal ID: open

ChemistryOpen

EISSN: 2191-1363
Publisher: Wiley

ARTICLE INFORMATION
==============================
PMCID: PMC3892191
PMID: 24551559
DOI: 10.1002/open.201380511
Article version: 1
Subjects: Graphical Abstract

Graphical Abstract: ChemistryOpen 5-6/2013

Print publication date: 2013 Dec
Electronic publication date: 2013 Dec 19
Volume: 2
Issue: 5-6
First page: 167
Last page: 170
Copyright: Copyright © 2013 WILEY-VCH Verlag GmbH & Co. KGaA, Weinheim
Copyright year: 2013
License: Re-use of this article is permitted in accordance with the Creative Commons Deed, Attribution 2.5, which does not permit commercial exploitation.
License URL: https://creativecommons.org/licenses/by/2.5/

==============================
ChemistryOPEN

Including Thesis Treasury

5-6/2013

ChemistryOPEN... is a multidisciplinary, gold-road, open-access, international forum for the publication of Full Papers and Communications from all areas of chemistry and related fields. *ChemistryOpen* also publishes the Thesis Treasury containing summaries of Ph.D. theses and links to the full version via our homepage. Based in Europe, *ChemistryOpen* attracts authors and readers from around the world, as open-access publishing becomes more important in all areas of chemistry. *ChemistryOpen* is co-owned by ChemPubSoc Europe and published by Wiley-VCH. Authors can submit their primary research articles and thesis summaries via our homepage by clicking “submit an article”. All contributions considered suitable for publication are subject to peer review, and if accepted, electronically processed and published online ensuring high quality and short publication times.

OPEN

Discover this journal online at:

WILEY ONLINE LIBRARY

wileyonlinelibrary.com

Most of the articles in this issue have already appeared online on wileyonlinelibrary.com. See http://www.chemistryopen.org under EarlyView®

### COVER PICTURE

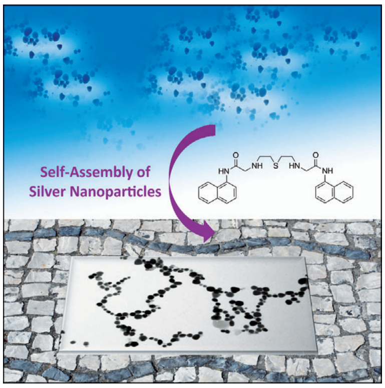

**The cover picture shows** the formation of one-dimensional nanochains of silver nanoparticles (AgNPs) upon addition of a polyamine molecular linker. In the present work, the binding ability of this linker was exploited, bearing different functional groups, which favors the self-assembly of silver and gold nanoparticles (AuNPs) into one-dimensional nanochains in aqueous solution. These nanochains visually resemble the artistic work of “calceteiros” (pavers) to build the “calçada Portuguesa” (Portuguese pavements) in Portugal (Photography “calçada Portuguesa”, Jose M. G. Pereira). Polyamine ligands are very versatile materials due to their water solubility and flexibility. The study of AgNPs and AuNPs in one-dimensional nanostructured formations are finally used as new sophisticated heavy metal ($Hg^{2+}$) ion chemosensors. For more details, see the Full Paper by Carlos Lodeiro, Christina Núñez and co-workers, on p. 200 ff.

### NEWS

Spotlights on our sister journals *172 – 175*

ChemistryOPEN Masthead

ChemPubSoc Europe www.chemistryopen.org

Austria

Belgium

Czech Republic

France

Germany

Greece

Hungary

Italy

Netherlands

Poland

Portugal

Slovakia

Spain

Sweden

Switzerland

### Editorial Advisory Board

### *Chairmen*

**Ramón Martínez-Máñez (Spain)**
**Thomas Wirth (UK)**

### *Honorary Chairman*

**Jean-Marie Lehn (France)**

### *Members*

Fernando Albericio (Spain)
Valentin Ananikov (Russia)
Didier Astruc (France)
Jochen Autschbach (USA)
Martin Banwell (Australia)
Vincenzo Barone (Italy)
F. Matthias Bickelhaupt (The Netherlands)
Kathrin Breuker (Austria)
Young Keun Chung (South Korea)
Richard G. Compton (UK)
Ulf Diederichsen (Germany)
Lothar Dunsch (Germany)
Antony Fairbanks (New Zealand)
Ernest Giralt (Spain)
Stefan Grimme (Germany)
Dirk Guldi (Germany)
Akira Harada (Japan)
Narayan Hosmane (USA)
Ken Houk (USA)
Osamu Ito (Japan)
Christoph Janiak (Germany)
Kazunori Kataoka (Japan)
Yoshiaki Kiso (Japan)
Mats Larhed (Sweden)
Lechoslav Latos-Grażyński (Poland)
Thisbe Lindhorst (Germany)
Luis M. Liz-Marzán (Spain)
Carlos Lodeiro (Portugal)
Todd L. Lowary (Canada)
Jean Martinez (France)
Pedro Matias (Portugal)
Stefan Matile (Switzerland)
Pedro Molina (Spain)
K. C. Nicolaou (USA)
Berit Olofsson (Sweden)
Oliver Reiser (Germany)
Kari Rissanen (Finland)
Knut Rurack (Germany)
James Rusling (USA)
Albert Schenning (The Netherlands)
Robert Schlögl (Germany)
Patrick Schmuki (Germany)
Ulrich S. Schubert (Germany)
Min Shi (China)
Norio Shibata (Japan)
Rint Sijbesma (The Netherlands)
Choong Eui Song (South Korea)
Petr Štěpnička (Czech Republic)
Brian Stoltz (USA)
Steve Suib (USA)
Reshef Tenne (Israel)
Matthew Todd (Australia)
Claudio Toniolo (Italy)
Jaume Veciana (Spain)
Hans-Achim Wagenknecht (Germany)
Ralph Weissleder (USA)
Gunther Wittstock (Germany)
Vivian W. W. Yam (China)
Chun-Hua Yan (China)
Jincai Zhao (China)

### Open Access and Copyright

All research articles published by *ChemistryOpen* are fully open access: immediately freely available to read, download and share. All *ChemistryOpen* articles are published under the terms of the Creative Commons Attribution Non-Commercial License which permits use, distribution and reproduction in any medium, provided the original work is properly cited and is not used for commercial purposes.

Copyright on any research article published by *ChemistryOpen* is retained by the author(s). Authors grant Wiley a license to publish the article and identify itself as the original publisher. Authors also grant any third party the right to use the article freely as long as its integrity is maintained and its original authors, citation details and publisher are identified.

### Use by commercial "for-profit" organisations

Use of Wiley Open Access articles for commercial, promotional, or marketing purposes requires further explicit permission from Wiley and will be subject to a fee. Copyright 2012 Wiley-VCH Verlag GmbH & Co. KGaA, Weinheim. All rights reserved. Requests to reproduce material from this journal content should be addressed to rights@wiley-vch.de, and reprints of *ChemistryOpen* articles can be purchased from chem-reprints@wiley-vch.de.

### Disclaimer

The Publisher and Editors cannot be held responsible for errors or any consequences arising from the use of information contained in this journal; the views and opinions expressed do not necessarily reflect those of the Publisher and Editors, neither does the publication of advertisements constitute any endorsement by the Publisher and Editors of the products advertised.

Wiley Open Access articles posted to repositories or websites are without warranty from Wiley of any kind, either express or implied, including, but not limited to, warranties of merchantability, fitness for a particular purpose, or non-infringement. To the fullest extent permitted by law Wiley disclaims all liability for any loss or damage arising out of, or in connection with, the use of or inability to use the content.

**Editors:**
Karen Hindson, Haymo Ross

**Deputy Editor:**
Natalia Ortúzar

**Associate Editor:**
Saskia Neubacher

**Secretary:**
Vanessa Müller

**Production:**
Stefanie Klein, Margitta Schmitt

**Telephone:**
(+ 49) 6201-606-305

**Fax:**
(+ 49) 6201-606-203

**E-mail:**
chemistryopen@wiley-vch.de

**Courier Services:**
Boschstrasse 12
69469 Weinheim (Germany)

**Regular Mail:**
Postfach 10 11 61
69451 Weinheim (Germany)

**Homepage:**
http://www.chemistryopen.org

**Manuscript Submission & Personal Homepage**
manuscriptXpress
**http://www.manuscriptxpress.com**

**Customer Services:**
www.wileycustomerhelp.com

**Marketing:**
Monika Silz (ext. 458, fax: ext. 100,
e-mail: msilz@wiley-vch.de)

**Advertising:**
Marion Schulz (ext. 565, fax: ext. 550,
e-mail: mschulz@wiley-vch.de, jspiess@wiley-vch.de)

**Copyright Permissions:**
Bettina Loycke (ext. 280, fax: ext. 447,
e-mail: rights@wiley-vch.de)

**Reprints, e-prints, posters:**
Carmen Leitner (fax: ext. 226)
e-mail: chem-reprints@wiley-vch.de

**Publishers:**
WILEY-VCH Verlag GmbH & Co. KGaA,
Postfach 10 11 61
69451 Weinheim (Germany)
http://www.wiley-vch.de/

**Publication:**
6 issues in 2013

© 2013 WILEY-VCH Verlag GmbH & Co. KGaA, Weinheim.

### COVER PROFILE

*"This collaboration started nearly 15 years ago with the purpose to use the expertise in the synthesis of polyamine acyclic and macrocyclic ligands and derivatives of the group at Santiago de Compostela, and the skills in nanochemistry and photophysical studies of the BIOSCOPE group for the development of new fluorescent nanostructured chemosensors."* More about the story behind the cover research can be found on p. 166.

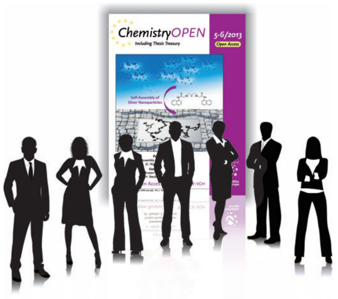

*A. Fernández-Lodeiro, J. Fernández-Lodeiro, C. Núñez,* R. Bastida, J. L. Capelo, C. Lodeiro**

***166***

**Polyamine Ligand-Mediated Self-Assembly of Gold and Silver Nanoparticles into Chainlike Structures in Aqueous Solution: Towards New Nanostructured Chemosensors**

### THESIS TREASURY

**Non-conventional coordination chemistry** occurs upon encapsulation of metal fragments in the cavity of a β-cyclodextrin-derived diphosphane (**WIDEPHOS**). It was found possible to form otherwise unstable metal species and slow down coordination processes, allowing the elucidation of novel mechanistic pathways for carbon–carbon bond-forming reactions.

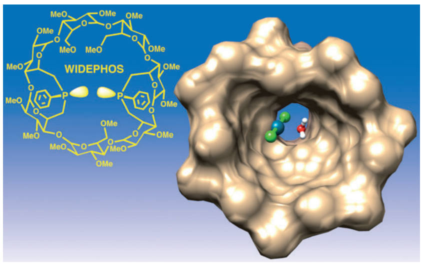

*R. Gramage-Doria**

***176 – 179***

**Metallocyclodextrins: Combining Cavitands with Metal Centres**

### COMMUNICATIONS

**Non-hinge binding:** A novel organometallic protein kinase inhibitor scaffold based on a cyclometalated 1,8-phenanthrolin-7(8*H*)-one ligand is reported, which binds to the ATP binding site of the protein kinase Pim1 in an unexpected, unusual non-ATP-mimetic fashion without mimicking the hydrogen-bonding interaction of the adenine nucleobase of ATP.

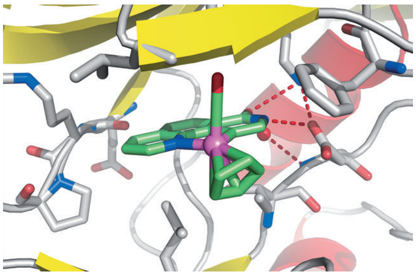

*K. Wähler, K. Kräling, H. Steuber,* E. Meggers**

***180 – 185***

**Non-ATP-Mimetic Organometallic Protein Kinase Inhibitor**

### FULL PAPERS

**Bonds and order:** The stability of the B-DNA double helix is shown (see illustration), based on dispersion-corrected density functional computations, to depend not only on the number of hydrogen bonds in Watson–Crick base pairs but also on the order and orientation in which these base pairs occur.

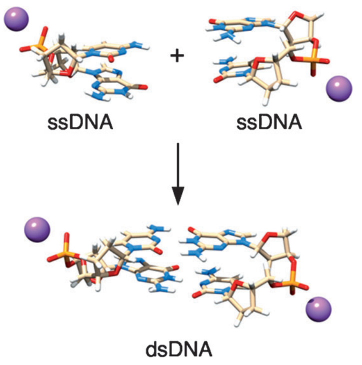

*G. Barone,* C. Fonseca Guerra, F. M. Bickelhaupt**

***186 – 193***

**B-DNA Structure and Stability as Function of Nucleic Acid Composition: Dispersion-Corrected DFT Study of Dinucleoside Monophosphate Single and Double Strands**

© 2013 Wiley-VCH Verlag GmbH & Co. KGaA, Weinheim

*A. Tilborg, S. Lanners, B. Norberg, J. Wouters**

**194 – 199**

**Solid-State-Trapped Reactive Ammonium Carbamate Self-Derivative Salts of Prolinamide**

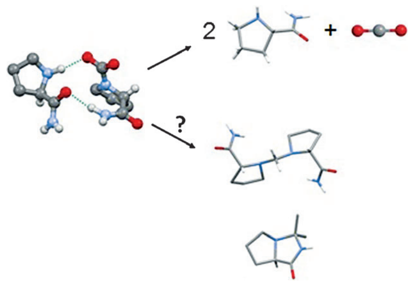

**Trapped within:** We report single-crystal characterization of polymorphs of the ammonium carbamate self-derivative salt of prolinamide, and the first crystal structure of prolinamide and of derived products. This study emphasizes the reactivity of carbonated amines in the solid state and opens perspectives for a systematic study of (solid-state) reactions involving these trapped reactive species.

*A. Fernández-Lodeiro, J. Fernández-Lodeiro, C. Núñez,* R. Bastida, J. L. Capelo, C. Lodeiro**

**200 – 207**

**Polyamine Ligand-Mediated Self-Assembly of Gold and Silver Nanoparticles into Chainlike Structures in Aqueous Solution: Towards New Nanostructured Chemosensors**

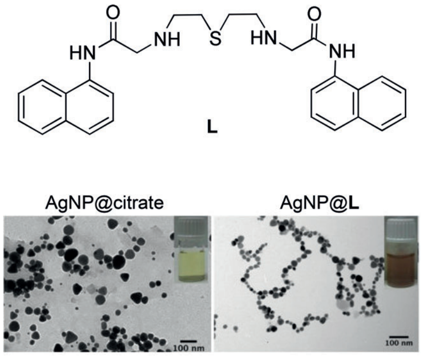

**1D Nanochain formation:** The binding ability of a polyamine molecular linker $(\mathbf{L})^{2-}$ bearing different functional groups, which favors the self-assembling of silver (AgNPs) and gold nanoparticles (AuNPs) into 1D nanochains in aqueous solution was explored. UV/Vis spectrophotometry and TEM were used to determine time-dependent structural changes associated with these 1D structure formations. Sensing of $Hg^{2+}$ using AgNPs@$(\mathbf{L})^{2-}$ and AuNPs@$(\mathbf{L})^{2-}$ assemblies was also carried out in aqueous solution.

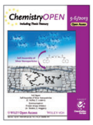

*Front Cover*

*L. L. Khemchyan, E. A. Khokhlova, M. M. Seitkalieva, V. P. Ananikov**

**208 – 214**

**Efficient Sustainable Tool for Monitoring Chemical Reactions and Structure Determination in Ionic Liquids by ESI-MS**

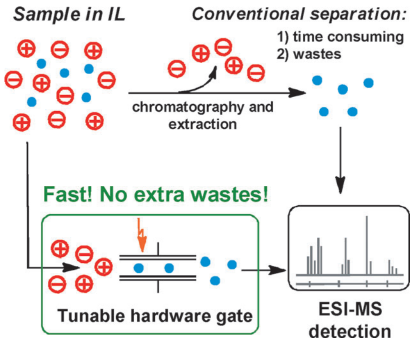

**Simple and effective:** A facile procedure for monitoring chemical reactions and characterizing compounds dissolved in ionic liquids is described using the MS/MS technique. Separation of species of interest was carried out using internal electronic gate available in electrospray mass spectrometers, instead of chromatography and extraction. The separation was carried out as a fast (milliseconds) and sustainable (no extra solvents) procedure.

* Author to whom correspondence should be addressed.

Supporting information on the WWW (see article for access details).

This is an open-access article, published under the terms and conditions of the Creative Commons Attribution Non Commercial License.

A video clip is available as Supporting Information on the WWW (see article for access details).

VIP Contributions labeled with this symbol have been judged as “Very Important Papers” by the referees.

### SERVICE

**All the Tables of Contents may be found on the WWW under: http://www.chemistryopen.org**

© 2013 Wiley-VCH Verlag GmbH & Co. KGaA, Weinheim
